# Detection of Zika Virus in *Aedes aegypti* and *Aedes albopictus* Mosquitoes Collected in Urban Forest Fragments in the Brazilian Amazon

**DOI:** 10.3390/v15061356

**Published:** 2023-06-12

**Authors:** Erika Oliveira Gomes, Lívia Sacchetto, Maurício Teixeira, Bárbara Aparecida Chaves, Adam Hendy, Claudia Mendonça, Izabele Guimarães, Ramon Linhares, Daniela Brito, Danielle Valério, Jady Shayenne Mota Cordeiro, Alexandre Vilhena Silva Neto, Vanderson Souza Sampaio, Vera Margarete Scarpassa, Michaela Buenemann, Nikos Vasilakis, Djane Clarys Baia-da-Silva, Maurício Lacerda Nogueira, Maria Paula Gomes Mourão, Marcus Vinícius Guimarães Lacerda

**Affiliations:** 1 Universidade do Estado do Amazonas (UEA), Manaus 69850-000, AM, Brazil; erika.gomes27@gmail.com (E.O.G.); djane.claryss@gmail.com (D.C.B.-d.-S.); mariapaula.mourao@gmail.com (M.P.G.M.); 2 Programa de Pós-Graduação em Medicina Tropical, PPGMT, Fundação de Medicina Tropical Dr. Heitor Vieira Dourado (FMT-HVD), Manaus 69040-000, AM, Brazil; maurili15@hotmail.com (M.T.); bachaves89@gmail.com (B.A.C.); claudiareism1@hotmail.com (C.M.); izabele.s.guimaraes@gmail.com (I.G.); ramonlinhares13@gmail.com (R.L.); brito.dani06@gmail.com (D.B.); dannyvalerio12@hotmail.com (D.V.); motajady8@gmail.com (J.S.M.C.); alexandre.neto94@gmail.com (A.V.S.N.); vandersons@gmail.com (V.S.S.); 3 Laboratório de Malária e Dengue, Instituto Nacional de Pesquisas da Amazônia (INPA), Manaus 69067-375, AM, Brazil; 4 Laboratório de Pesquisa em Virologia, Faculdade de Medicina de São José do Rio Preto (FAMERP), São José do Rio Preto 15090-000, SP, Brazil; liviasacchetto@gmail.com (L.S.); mauricio.nogueira@edu.famerp.br (M.L.N.); 5 Department of Pathology, University of Texas Medical Branch, Galveston, TX 77555, USA; adhendy@utmb.edu (A.H.); nivasila@utmb.edu (N.V.); 6 Instituto Todos pela Saúde (ITpS), São Paulo 01310-942, SP, Brazil; 7 Laboratório de Genética Populacional e Evolução de Mosquitos Vetores da Malária e Dengue, Instituto Nacional de Pesquisas da Amazônia (INPA), Manaus 69067-375, AM, Brazil; vmscarpassa@gmail.com; 8 Department of Geography, New Mexico State University, Las Cruces, NM 88003, USA; elabuen@nmsu.edu; 9 Center for Vector-Borne and Zoonotic Diseases, University of Texas Medical Branch, Galveston, TX 77555, USA; 10 Center for Biodefense and Emerging Infectious Diseases, University of Texas Medical Branch, Galveston, TX 77555, USA; 11 Center for Tropical Diseases, University of Texas Medical Branch, Galveston, TX 77555, USA; 12 Institute for Human Infection and Immunity, University of Texas Medical Branch, Galveston, TX 77555, USA; 13 Instituto Leônidas & Maria Deane, ILMD/FIOCRUZ Amazonia, Manaus 69057-070, AM, Brazil; 14 Programa de Pós-Graduação em Assistência Farmacêutica, Universidade Federal do Amazonas (UFAM), Manaus 69080-900, AM, Brazil; 15 Departamento de Ensino e Pesquisa, Universidade Nilton Lins, Manaus 69058-030, AM, Brazil

**Keywords:** mosquito surveillance, arbovirus, forest, Amazon, ZIKV

## Abstract

Zika virus (ZIKV) is an RNA flavivirus (*Flaviviridae* family) endemic in tropical and subtropical regions that is transmitted to humans by *Aedes* (*Stegomyia*) species mosquitoes. The two main urban vectors of ZIKV are *Aedes aegypti* and *Aedes albopictus,* which can be found throughout Brazil. This study investigated ZIKV infection in mosquito species sampled from urban forest fragments in Manaus (Brazilian Amazon). A total of 905 non-engorged female *Ae. aegypti* (22 specimens) and *Ae. albopictus* (883 specimens) were collected using BG-Sentinel traps, entomological hand nets, and Prokopack aspirators during the rainy and dry seasons between 2018 and 2021. All pools were macerated and used to inoculate C6/36 culture cells. Overall, 3/20 (15%) *Ae. aegypti* and 5/241 (2%) *Ae. albopictus* pools screened using RT-qPCR were positive for ZIKV. No supernatants from *Ae. aegypti* were positive for ZIKV (0%), and 15 out of 241 (6.2%) *Ae. albopictus* pools were positive. In this study, we provide the first-ever evidence of *Ae. albopictus* naturally infected with ZIKV in the Amazon region.

## 1. Introduction

Zika virus (ZIKV) is an RNA flavivirus (genus *Flavivirus*, family *Flaviviridae*) endemic in tropical and subtropical regions that is transmitted to humans by *Aedes* (*Stegomyia*) species mosquitoes [1]. ZIKV was first isolated in 1947 from a sentinel *Rhesus macaque* in the Ziika forest in Uganda [2] and soon after from a pool of arboreal *Aedes africanus* mosquitoes collected in the same area [2]. However, the first notable ZIKV outbreak in humans was reported in 2007 on Yap Island in the Federated States of Micronesia [3]. Subsequently, ZIKV crossed the Pacific and entered the Americas via Brazil in 2013 [4]. The severity of the disease was only recognized during the ensuing epidemic, when it was associated with Guillain–Barré syndrome in adults and congenital Zika Syndrome in infants born from ZIKV-infected mothers [5]. Due to the absence of specific treatments for the disease and an increase in cases in the Americas, the World Health Organization declared the ZIKV outbreak a public health emergency of international concern (PHEIC) in February 2016 [6].

The two main urban vectors of ZIKV are container-breeding *Ae. aegypti* and *Ae. albopictus* mosquitoes [7]. Both thrive in highly populated urban environments where they may also transmit dengue and chikungunya [8]. *Ae. aegypti* is highly anthropophilic and considered to be the principal vector of ZIKV in urban and peri-urban areas. This may be related to genetic (which defines anthropophilic) and environmental (related to the presence of hosts) factors affecting its distribution and vector competence in natural populations [9]. *Ae. albopictus* is more likely to be found in rural areas [10]. Several studies have detected mixed human–animal blood meals in *Ae. albopictus*, highlighting its potential to serve as a bridge vector for zoonotic pathogens in rural settings [11,12]. Both species have high potential for geographic expansion driven by increased global trade and travel [13] and climate change [2,14]. Mapping the local and global distribution of these vectors and the geographic determinants of their ranges is essential for planning vector and pathogen surveillance, carrying out public health responses, and controlling arbovirus transmission [13,15].

Brazil is home to many primate species, some of which are known to be susceptible to ZIKV infection [16], and sylvatic mosquitoes, including *Haemagogus* and *Sabethes* species, which are potentially capable of transmitting the virus [17]. Given the presence of potential animal reservoirs of disease and sylvatic vectors, it is possible that ZIKV has spilled back into wild areas since being introduced to the neotropics [18].

Until now, there has been no evidence for the circulation of ZIKV among *Ae. aegypti* and *Ae. albopictus* sampled from forest fragments in the Brazilian Amazon, which would heighten interest in their role as potential bridge vectors. In Brazil, ZIKV has only been detected in *Ae. albopictus* collected in urban and peri-urban areas of Espírito Santo [19], Rio de Janeiro [20], São Paulo [21], and Mato Grosso [22]. This study investigated ZIKV infection in *Ae. aegypti* and *Ae. albopictus* mosquitoes sampled at the urban–forest interface in Manaus, the capital of Amazonas State, Brazil. The city has more than two million inhabitants [23], and 447 confirmed Zika human cases were reported in Manaus between March 2018 and June 2021 [24].

## 2. Materials and Methods

**Figure 1:**
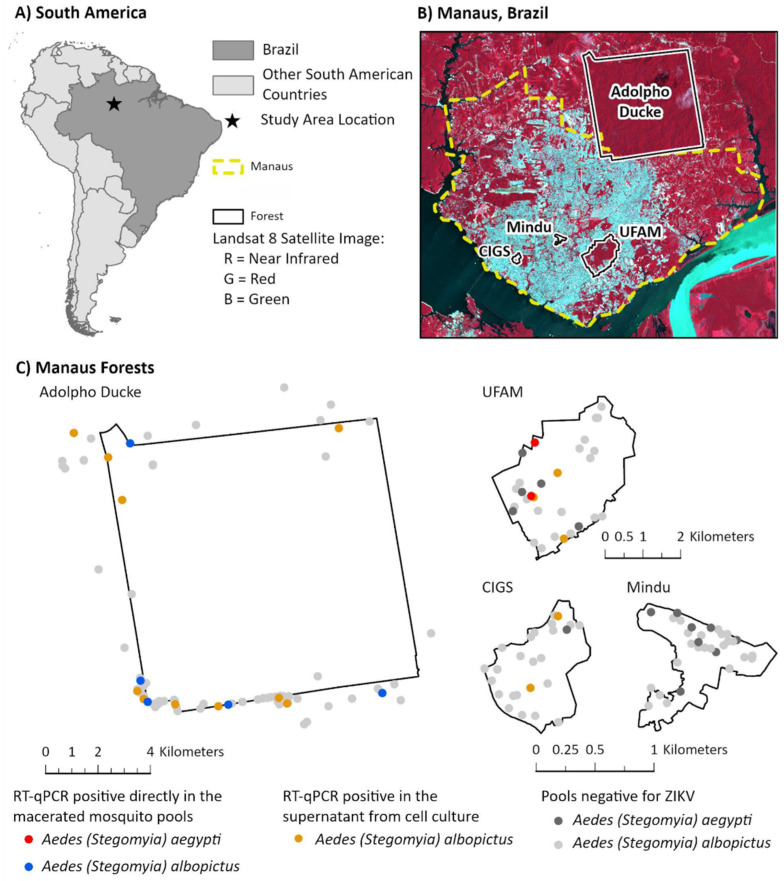
Location of sampled mosquitoes. (**A**) Geopolitical map of South America, highlighting Brazil and study area location. (**B**) Satellite image infrared composite of Manaus city and forest boundaries. 1. Zoológico do Centro de Instrução de Guerra na Selva (CIGS), 2. Parque Municipal do Mindu, 3. Universidade Federal do Amazonas (UFAM), and 4. Reserva Florestal Adolpho Ducke. (**C**) Manaus forests showing a spatial distribution of sampled mosquitoes and positive pools. Red and blue dots represent RT-qPCR-positive specimens detected directly from the macerated mosquito pools. Orange dots show RT-qPCR-positive results in the supernatant from cell culture. Dark and light gray dots show pools of *Ae. aegypti* and *Ae. albopictus*, respectively, that tested negative for ZIKV. Maps were created using ArcGIS Pro.

Mosquitoes were mostly sampled in the rainy season and occasionally in the dry season from March 2018 to June 2021 in four forest fragments located within and on the edge of Manaus (Figure 1): 1. Centro de Instrução de Guerra na Selva (CIGS, 6000 m^2^, 3.101172° S, 60.044781° W), 2. Parque Municipal do Mindu (Mindu, 4800 m^2^, 3.080594° S, 60.004367° W), 3. Universidade Federal do Amazonas (UFAM, 6700 m^2^, 3.100422° S, 59.976517° W), and 4. Reserva Florestal Adolpho Ducke (Ducke, 100,000 m^2^, 2.94890° S, 59.92992° W). These collection areas and periods were also evaluated in previous studies by Hendy et al. [25,26,27,28]. The rainy season lasts from about November to May, while the dry season lasts from about June to October [27].

Each forest fragment was stratified according to its distance from the forest edge to the interior and according to Normalized Difference Vegetation Index (NDVI) as previously described [25]. Mosquitoes were generally sampled four days per week at sites distributed within these strata as part of an ongoing project investigating mosquito communities at urban–forest edges [25,27]. Sampling methods included BG-Sentinel traps baited with/without dry ice (CO_2_) and a BG-Lure (Biogents AG, Regensburg, Germany), Prokopack aspirators (John W. Hock Company, Gainesville, FL, USA), and entomological hand nets (Supplementary Table S1). Sampled mosquitoes were transferred to a −80 °C freezer immediately upon returning from the field. Individual mosquitoes were identified on a chill table (BioQuip, Rancho Dominguez, CA, USA) using a stereomicroscope and relevant taxonomic keys [29]. Non-engorged female *Ae. aegypti* and *Ae. albopictus* specimens were grouped in pools of up to 20 mosquitoes according to date, collection site, and species. Pools of mosquitoes were macerated in 1 mL of phosphate-buffered saline using a cell homogenizer (Kimble & Chase^®^, model 749540-0000, NJ, USA) and centrifuged at 10,000 rpm for 5 min. All macerated pools were filtered using 0.22 µm filters attached to a 3 mL syringe. Two evaluations using RT-qPCR were performed in parallel, namely, one directly on macerated mosquito pools, and one on supernatant from cell culture, as ZIKV does not lead to cytopathic changes. Positivity of supernatant from the macerated pools inoculated into C6/36 cells indicated viral replication and viability.

Total RNA was extracted from 140 µL of macerated mosquitos using QIAamp Viral RNA mini kit (Qiagen, Hilden, Germany) according to the manufacturer’s protocol. RT-qPCR was then performed with primers targeting the flavivirus NS5 gene [30] (Supplementary Table S2) using the Transcriptor One-Step RT-qPCR kit (Roche Diagnostics, Mannheim, Germany) according to the manufacturer’s instructions. Samples were considered positive when presenting a peak with a melting temperature (Tm) between 80–84 °C. RNA from positive samples was examined using specific primers and probes for dengue virus serotypes 1, 2, 3, and 4 (DENV 1-4) [31]; ZIKV [32]; and yellow fever virus (YFV) [33]. In parallel, 100 µL of macerate of each mosquito pool was inoculated onto 24-well plates containing C6/36 cells maintained in Leibowitz L-15 culture medium plus 10% inactivated fetal bovine serum and 1% penicillin–streptomycin [34]. The cell culture was maintained and observed for 7 days in an incubator at 28 °C. RNA extraction and RT-qPCR were then performed following the above protocols. ZIKV RNA from a positive patient (Ct 15) and nuclease-free water were used as positive and negative controls in all RT-qPCR reactions, respectively. Samples were considered positive when they amplified with a cycle threshold (Ct) value of 38 [32].

## 3. Results and Discussion

A total of 1316 *Ae. aegypti* and *Ae. albopictus* mosquitoes were collected during the study. Of these, 22/43 of the *Ae. aegypti* specimens (51.2%) and 883/1273 of the *Ae. albopictus* specimens (69.4%) were female. The former species was only present in low relative abundance in two-thirds of the forest fragments sampled within the urban matrix, while the latter was found in all forest fragments including the large Ducke reserve on the edge of the city (Figure 1). ZIKV was detected in 3/20 (15%) *Ae. aegypti* pools and 5/241 (2%) *Ae. albopictus* pools screened using RT-qPCR. Inoculation of C6/36 cells with the macerate showed that 0/20 *Ae. aegypti* and 15/241 (6.2%) *Ae. albopictus* pools were positive. The relative distribution of ZIKV positive pools among the forest fragments is shown in Figure 1 and Table 1. All pools tested negative for DENV and YFV.

Our findings show a high percentage of pools that tested positive for ZIKV based on mosquitoes collected in three of the four forest fragments studied in Manaus. High pool positivity rates for this pathogen have been observed elsewhere in Brazil [21,22,35]. Ayllón et al. [35] conducted a surveillance program for mosquito-borne viruses from February 2014 to June 2016 and detected ZIKV in two pools (1.1%) of engorged *Ae. aegypti* females out of 178 (predominantly *Ae. aegypti*) pools that were screened from Rio de Janeiro. Parra et al. [21] conducted mosquito-based Zika virus surveillance from 2015 to 2018 and reported ZIKV infection in 55/607 (9.1%) of the pools of *Ae. aegypti* females and in 1/11 (9.1%) of the pools of *Ae. Albopictus* females collected in a suburban neighborhood of São José do Rio Preto, São Paulo State. Additionally, Neves et al. [22] searched for arboviruses in mosquito body pools sampled in southern Mato Grosso during the rainy season of 2018 at 21 bird-watching sites. They detected ZIKV RNA in pools of females and males of both species, with 11/77 (14.3%) *Ae. Aegypti* and 5/48 (10.4%) *Ae. Albopictus* pools testing positive.

In this study, we present the first-ever evidence of *Ae. albopictus* naturally infected with ZIKV in the Amazon region, which was based on collections made in forest fragments embedded within urban and peri-urban areas of the city. Hendy et al. [28] have shown overlapping distributions of urban and forest mosquito species, including known virus vectors, at the edges of the same areas, highlighting the risk of arbovirus exchange through multiple bridge vectors. In these settings, there is a high risk for interaction between adjacent residents, urban and sylvatic mosquitoes, and non-human primates such as *Saguinus bicolor* [36], which may affect the dynamics of transmission and dispersion of arboviruses [36,37]. Therefore, it is important that the systematic monitoring and screening of arthropod vectors is carried out to allow for better assessments of the risk of zoonotic pathogen exchange and to improve planning and guidance for arbovirus and vector control measures.

This study has some limitations. Engorged specimens were not analyzed, and individual mosquito parity was not evaluated. Estimating parity rates provides information about the physiological age of mosquito populations, which can be useful if comparing spatial and temporal infection rates within and between studies or identifying periods of high transmission risk. We only encountered small numbers of *Ae. aegypti* since the sampled sites were situated in forested areas, where this species is seldom found, and could not compare infection rates with *Ae. Albopictus*, which was present in higher relative abundance as reported elsewhere [25,28]. However, we cannot exclude its possible role as a bridge vector since *Ae. aegypti* has been found in forest fragments in Manaus and elsewhere in Brazil.

We detected the presence of *Ae. aegypti* and *Ae. albopictus* naturally infected with ZIKV in forest fragments in the Brazilian Amazon. Our results provide a better understanding of ZIKV vectors at urban–forest edges and the mechanisms by which zoonotic arboviruses may emerge. Importantly, our findings may contribute to the development of risk models that help predict the emergence of pathogens with the potential to cause devastating outbreaks. The establishment of data-driven surveillance networks for the early detection of arbovirus transmission is critical [38] and may help mitigate or manage future disease outbreaks, but it will require extensive collaboration between health services, researchers, and environmental authorities.

## Figures and Tables

**Table 1:** Profiles of mosquito pools that tested positive for ZIKV infection.

Location	Collection Method	Date Collected (M/D/Y) *	Distance (m)	** NDVI	Species	Number of Mosquitoes per Pool	Ct of ZIKV-Positive Samples in C6/36	Ct of ZIKV-Positive Samples in Mosquito Macerate
1	BG-Sentinel	05/10/2018	0	Low	*Aedes albopictus*	9	33.41	*** N
1	Aspirator	07/16/2018	50	Low	*Aedes albopictus*	2	35.23	N
3	BG-Sentinel	04/26/2018	0	Medium	*Aedes albopictus*	1	33.96	N
3	BG-Sentinel	06/12/2018	0	Low	*Aedes albopictus*	10	36.3	N
3	BG-Sentinel	08/22/2018	500	low	*Aedes albopictus*	1	33.75	N
3	BG-Sentinel	04/12/2018	50	Low	*Aedes aegypti*	1	N	35.6
3	BG-Sentinel	05/23/2018	0	High	*Aedes aegypti*	1	N	35.4
4	BG-Sentinel	01/15/2019	0	Medium	*Aedes albopictus*	2	N	35.1
4	Aspirator	02/01/2019	0	Medium	*Aedes albopictus*	3	34.42	N
4	BG-Sentinel	02/01/2019	0	Low	*Aedes aegypti*	1	N	37.5
4	BG-Sentinel	02/01/2019	0	Medium	*Aedes albopictus*	3	33.21	N
4	BG-Sentinel	02/05/2019	0	Medium	*Aedes albopictus*	1	33.39	N
4	Net	02/07/2019	0	High	*Aedes albopictus*	2	37.2	N
4	Net	04/09/2019	500	Low	*Aedes albopictus*	1	34.78	N
4	Net	05/06/2019	0	Low	*Aedes albopictus*	1	N	33.8
4	BG-Sentinel	05/28/2019	0	Low	*Aedes albopictus*	2	N	34.8
4	Net	06/10/2019	0	Low	*Aedes albopictus*	1	N	35.1
4	BG-Sentinel	12/06/2019	0	Medium	*Aedes albopictus*	1	N	35.9
4	BG-Sentinel	01/24/2020	0	Low	*Aedes albopictus*	1	33.85	N
4	Aspirator	02/28/2020	0	Low	*Aedes albopictus*	1	36	N
4	Net	05/18/2021	0	High	*Aedes albopictus*	1	37.5	N
4	Net	06/02/2021	0	High	*Aedes albopictus*	1	36.9	N
4	Net	06/09/2021	0	Low	*Aedes albopictus*	3	36.4	N

* M/D/Y: Month/Day/Year. ** NDVI values within these forest areas calculated using an equal frequency classification as either low (data range 0.472–0.859), medium (0.860–0.869), or high (0.870–1.000) NDVI. *** N: negative.

## Data Availability

The authors declare that the data supporting the findings of this study are available within the article.

## Supplementary Materials

The following supporting information can be downloaded at: https://www.mdpi.com/article/10.3390/v15061356/s1.
